# Complex Ecotype Dynamics Evolve in Response to Fluctuating Resources

**DOI:** 10.1128/mbio.03467-21

**Published:** 2022-05-16

**Authors:** Megan G. Behringer, Wei-Chin Ho, John C. Meraz, Samuel F. Miller, Gwyneth F. Boyer, Carl J. Stone, Meredith Andersen, Michael Lynch

**Affiliations:** a Department of Biological Sciences, Vanderbilt University, Nashville, Tennessee, USA; b Department of Pathology Microbiology and Immunology, Vanderbilt University Medical Center, Nashville, Tennessee, USA; c Evolutionary Studies Initiative, Vanderbilt University, Nashville, Tennessee, USA; d Biodesign Center for Mechanisms of Evolution, Arizona State University, Tempe, Arizona, USA; Pacific Northwest National Laboratory

**Keywords:** black queen hypothesis, experimental evolution, intraspecific cooperation, *Escherichia coli*, starvation

## Abstract

Ecotypic diversification and its associated cooperative behaviors are frequently observed in natural microbial populations whose access to resources is often sporadic. However, the extent to which fluctuations in resource availability influence the emergence of cooperative ecotypes is not fully understood. To determine how exposure to repeated resource limitation affects the establishment and long-term maintenance of ecotypes in a structured environment, we followed 32 populations of Escherichia coli evolving to either 1-day or 10-day feast/famine cycles for 900 days. Population-level analysis revealed that compared to populations evolving to 1-day cycles, 10-day populations evolved increased biofilm density, higher parallelism in mutational targets, and increased mutation rates. As previous investigations of evolution in structured environments have identified biofilm formation as the earliest observable phenotype associated with diversification of ecotypes, we revived cultures midway through the evolutionary process and conducted additional genomic, transcriptional, and phenotypic analyses of clones isolated from these evolving populations. We found not only that 10-day feast/famine cycles support multiple ecotypes but also that these ecotypes exhibit cooperative behavior. Consistent with the black queen hypothesis, or evolution of cooperation by gene loss, transcriptomic evidence suggests the evolution of bidirectional cross-feeding behaviors based on essential resources. These results provide insight into how analogous cooperative relationships may emerge in natural microbial communities.

## INTRODUCTION

Present in every colonizable habitat, microbes are the most resilient and ubiquitous organisms on the planet. This resilience is often attributed to the vast metabolic diversity present in microbial communities that allows these communities to withstand harsh conditions, such as prolonged resource limitation and rapid environmental disruptions. Although this metabolic diversity is often investigated on the species level by describing the roles of diverse taxa occupying a defined habitat, or microbiome, it has become clear that metabolic diversity can also evolve within a taxon and result in distinct subpopulations, or ecotypes. Microbiologists’ understanding of how ecotypic diversity evolves has been guided primarily by allopatric models, such as geographical or chemical barriers in a habitat (1) and spatially heterogeneous environments (2). However, more recently, studies describing sympatric diversification in relatively simple environments have drawn attention, as the metabolism of primary resources can result in the production of metabolic waste products, which make the environment much more complex (3–6). For example, the diversification and coexistence of ecotypes have been found in multiple cases of experimental evolution of Escherichia coli in glucose-limited media (7–10). In these cases, the evolution of incidental, or one-way, cross-feeding on metabolic waste products underlies ecotypic diversification, and the coexistence of the resulting ecotypes is thought to be maintained by negative frequency-dependent selection (11–14).

Despite one-way cross-feeding repeatedly emerging in experimentally evolved cultures, the evolution of mutualistic, or bidirectional, cross-feeding is less commonly observed. In theory, the evolution of cooperative cross-feeding behaviors in a microbial population depends on the complementary changes of metabolic abilities between ecotypes and their combined fitness consequences, as the resulting combined fitness advantages can stabilize the cross-feeding interactions (15, 16). These cross-feeding interactions can be further stabilized by gene loss, forcing the ecotypes to be codependent (17, 18) in a manner described by the black queen hypothesis. Specifically, the black queen hypothesis proposes that reductive evolution involving the loss of biosynthetic abilities may provide benefits to the individual, as producing fewer metabolites and expressing fewer proteins potentially can save energy (19). However, a loss of metabolic ability could also be the product of neutral accumulation of degenerative mutations (20). Therefore, assessing the fitness of cross-feeders, in isolation and in the presence of their cross-feeding partners, will help us to better understand the evolutionary dynamics involved in the emergence of intraspecific metabolic diversity in microbial communities.

Environmental and genetic context can also potentially affect the likelihood of ecotypic diversification and the emergence of cross-feeding behaviors. Microbes frequently encounter environments with fluctuating resource availability (21, 22). For example, as an opportunistic pathogen, E. coli has a broad habitat ranging from soil and wastewater to the lower gut, often oscillating between feast and famine (23–25). Of the conditions encountered during feast/famine fluctuations, starvation has been observed to encourage diversification (26, 27). However, it is less known how diversity is affected by environmental disruptions, such as the rapid replenishment of resources during feast/famine cycles. It has also been suggested that plasticity in the induction of stress responses can induce changes in molecular phenotypes such as mutation rates (28–30). If the evolution of cross-feeding depends on the spontaneous introduction of rare beneficial mutations, a higher mutation rate may facilitate efficiency in exploring the fitness landscape and rapid establishment of ecotypes due to the arrival of diverged lineages at isolated adaptive peaks (31–33). Thus, it is of particular interest to test how microbial populations respond to combinations of different feast/famine cycles and initial base genetic mutation rates.

To study the effect of different feast/famine cycles on the evolution of diversity in microbial populations, we experimentally evolved E. coli in culture tubes containing LB broth under two different feast/famine cycle conditions: fresh LB broth supplied every 1 and 10 days. In addition, to understand how differences in genetic mutation rates further affect evolutionary dynamics, we utilized ancestral lines with two initial genetic backgrounds: a wild-type (WT) strain and a WT-derived strain with impaired methyl-directed mismatch repair (MMR^−^; yielding an ~150× increase in the single nucleotide mutation rate by deleting *mutL*) (34). Each combination of genetic background and feast/famine cycle condition was replicated in eight parallel replicates, which resulted in 32 experimental populations (8 × 2 × 2). Polymorphism data from periodic whole-genome sequencing allowed a general survey of subpopulation structure, parallelism within each treatment combination, and divergence between treatments. We also quantified traits that may have distinct patterns in evolutionary responses to feast/famine cycles, including biofilm formation and mutation rates. Using genomic and transcriptomic data of multiple single clones from the same population, we further proposed a detailed model on the emergence of cross-feeding and ecotypic diversification.

## RESULTS

### Ten-day feast/famine cycles support the long-term coexistence of ecotypes.

Experimental populations of Escherichia coli were evolved in a complex environment consisting of 10 mL of LB broth in 16- by 100-mm glass culture tubes. Our 32 populations were split evenly by genetic background (WT versus MMR^−^) and transfer interval (cycles of 1 day versus 10 days), with each transfer resulting in a 1:10 dilution of the evolving population. Genomic evolution was assessed every 100 days via shotgun metagenomic sequencing of DNA collected from 1 mL of pretransfer culture. After 900 days of evolution, we examined how increased intervals of starvation between feedings affect the intrapopulation diversification and the evolution of long-term coexisting ecotypes.

**FIG 1:**
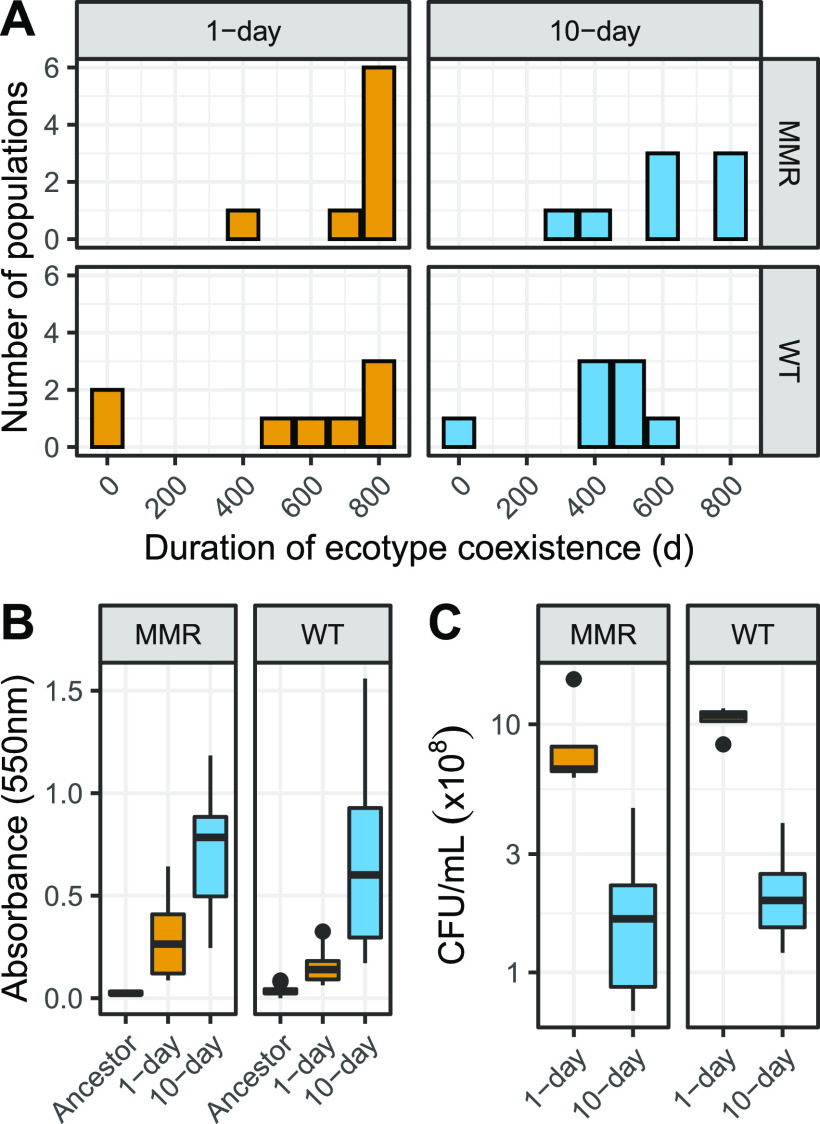
Presence of coexisting ecotypes is supported by genomic data and is associated with increased biofilm formation. (A) Distribution of durations of coexistence of major and minor clades, or multiple ecotypes, as determined by the caHMM among the parallel experimental populations for each resource limitation cycle/genetic background combination for 1 day (orange) or 10 days (blue). (B) Quantification of biofilm formation for the ancestral clone and evolved populations at the 900-day time point. Biofilm was stained with crystal violet and measured at an absorbance of 550 nm via microplate reader after 24 h of static growth in a 96-well plate. (C) Quantification of CFU numbers for the evolved populations after 24 h of growth. The observed increases of biofilm formation in populations evolving to 10-day feast/famine cycles cannot be explained by population-size differences, as these populations exhibit lower CFU counts after 24 h of growth. For panels B and C, boxplots visualize the quantile distribution of measurements.

As the 1-day culture environment is known to promote ecotypic diversification characterized by long-term coexistence of two diverging clades (35), we applied a clade-aware hidden Markov model (caHMM) to our time-series metagenomic sequencing data to determine if 10-day feast/famine cycles similarly support the long-term presence of multiple ecotypes (9). Briefly, caHMM assumes coexistence of two clades (major and minor) to infer whether each mutation belongs to the basal clade (i.e., mutations that sweep through the entire population before the establishment of subpopulations), the major clade, or the minor clade and infer if a mutation is fixed or polymorphic within its assigned clade throughout the course of experimental evolution. We defined the duration of coexistence for two ecotypes in a population by the maximum interval in which both major and minor clades are detected by caHMM. Overall, MMR^−^ populations maintained multiple ecotypes longer than WT populations, with no difference in ecotype coexistence between 1-day and 10-day feast/famine cycles (*P*_WT vs MMR-_ = 0.014; *P*_1 day vs 10 day_ = 0.136; analysis of variance [ANOVA] with Tukey’s honestly significant difference [HSD]) (Fig. 1A). Therefore, 10-day feast/famine cycles also have a similar tendency to support intraspecific diversification.

Phenotypically, the formation of biofilms at the surface-air interface is an observation associated with diversification (35) and has been observed in the culture of E. coli (35) and other microbial organisms in structured environments (1). In response to starvation conditions, microbes often respond by dispersing and initiating detachment from biofilm structures (36–38). However, recent investigation of heterogeneity in biofilms has suggested that carbon starvation can select for dispersal-insensitive mutants (39). To determine if repeated conditioning to longer starvation conditions affects the evolution of E. coli biofilms, we quantified biofilm density in 96-well plates after 24 h of static growth. In both WT and MMR^−^ populations, biofilms evolved to be significantly thicker in populations evolved to 10-day feast/famine cycles (*P*_WT_ = 2.6 × 10^−8^; *P*_MMR-_ = 6.4 × 10^−11^) (Fig. 1B). These increases in biofilm density are unlikely to be explained by evolved increases in carrying capacity, as populations evolving to 1-day transfer cycles reach higher total population sizes than populations evolving to 10-day cycles (*P*_WT_ = 1.7 × 10^−6^; *P*_MMR-_ = 7.9 × 10^−4^) (Fig. 1C).

### High mutational parallelism despite increased mutation rates in 10-day feast/famine cycles.

**FIG 2:**
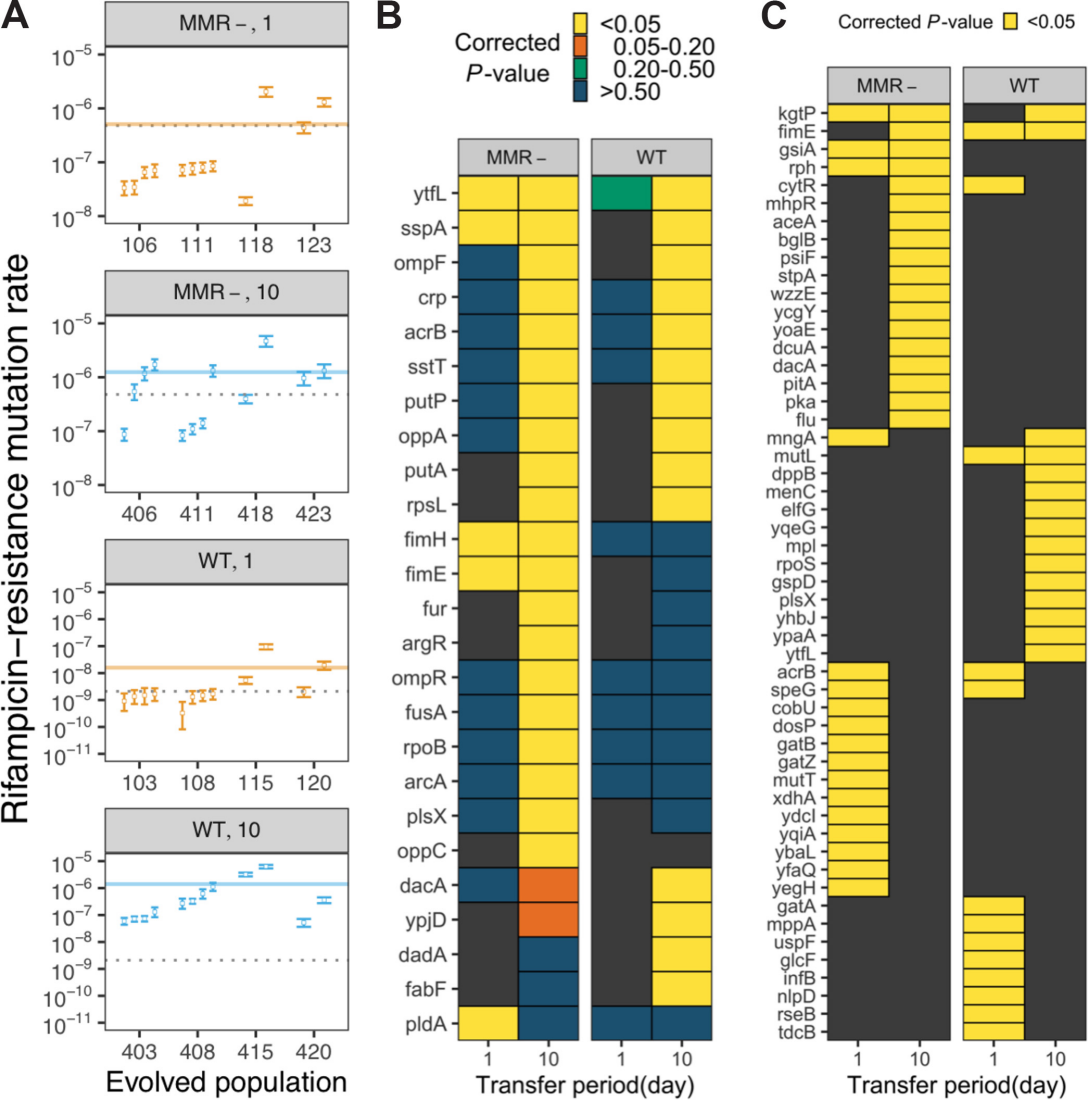
Genomic responses to 10-day feast/famine cycles include increased base genetic mutation rates and high parallelism in fixed mutations within ecotypes. (A) Mutation rates of populations at the 900-day time point for each resource limitation cycle (1 or 10 days) and genetic background (MMR^−^ or WT) as measured by fluctuation tests. In each combination, four evolved replicate lines were tested by isolating and measuring two or four clones per evolved line. As mutation rates are estimated via maximum likelihood, the open circle and error bar represent the mean and the 95% confidence interval for each clone. The gray dashed line represents the mutation rate measurement of the corresponding ancestor. The transparent colored lines represent the mean mutation rate measurement of each combination. (B and C) Heatmaps visualize genes that are likely under positive selection, as they are enriched for nonsynonymous mutations that significantly contribute to the increased sum of *G* scores (B) or significantly overrepresented structural mutations (IS element insertions and indels) (C). Significance levels (simulated *P* values with Bonferroni correction) are shown by the different nonblack colors of tiles. Genes with no such hits in a particular resource limitation cycle/genetic background combination are shown by black tiles.

Prior studies have suggested that starvation-associated stress temporarily increases mutation rates (28). These temporary increases can also become permanent, as E. coli populations subjected to extreme durations of starvation have repeatedly evolved increased base genetic mutation rates due to mutations in the methyl-directed mismatch repair pathway (30). To determine the effects of 10-day feast/famine cycles on base genetic mutation rates, we performed fluctuation tests for rifampin resistance (40) on clones isolated from a subset of four evolved populations from each genetic background and feast/famine treatment (16 populations total). For each clone, the 95% confidence interval of mutation rate was estimated and used for testing whether there is a significant difference from the ancestor. For WT backgrounds, all 12 clones isolated from 10-day populations exhibited significantly higher rates of rifampin resistance than the WT ancestor (Fig. 2A). In contrast, only 3 of 12 clones from 1-day WT populations show significant mutation rate increases. Increases in basal mutation rate were also observed in the MMR^−^ backgrounds; 6 of 12 clones isolated from 10-day populations evolved higher rifampin resistance rates than the MMR^−^ ancestor, but only 2 of 12 clones isolated from 1-day populations exhibit similar rifampin resistance rate increases. The estimated basic mutation rates of the 1-day clones are also significantly lower than those for the 10-day clones (WT, *P* = 9 × 10^−6^; MMR^−^, *P* = 6 × 10^−3^, Mann–Whitney *U* test). Thus, widespread evolution of mutator alleles is common in response to 10-day feast/famine cycles.

Increased mutation rates can contribute to adaptive diversification by fueling the exploration of adaptive landscapes and providing the genetic variation necessary for the evolution of specialized ecotypes. Concurrently, repeated strong selection can result in the rapid fixation of mutator alleles due to genetic hitchhiking (41), as the probability that a mutator allele fixes increases with the number of adaptive mutations needed to thrive in an environment (42). By calculating the extent of parallel evolution between populations in each evolutionary treatment using two metrics (sum of *G* scores [43] and mean Bray-Curtis similarity [44]), we next examined how 10-day feast/famine cycles influence which genes represent adaptive targets and whether or not increased mutation rates are associated with an increase in the number of adaptive targets that exhibit high mutational parallelism (45). Conservatively, we focused on the nonsynonymous mutations that were inferred to be fixed within either the basal, major, or minor clade of each evolving population by caHMM. If fixed nonsynonymous mutations are concentrated in a smaller subset of genes within a genetic background and feast/famine treatment combination, then the resulting values for these two metrics will be larger. For all treatment combinations, both the sum of *G* scores and the Bray-Curtis similarity values were significantly greater than expected based on a simulated null distribution (see Fig. S1A and B in the supplemental material), revealing a significant amount of parallel evolution within treatments and suggesting that positive selection is shaping these populations. Interestingly, the statistical significance of both metrics for 10-day populations is much greater than that for 1-day populations, suggesting that 10-day populations have a smaller pool of adaptive targets while requiring a greater number of adaptive mutations to thrive in the harsher 10-day feast/famine environment.

10.1128/mbio.03467-21.1 FIG S1 Evolutionary parallelism for each combination of feast/famine cycle and genetic background. (A) Within each combination of feast/famine cycle (1 day, orange; 10 days, blue) and genetic background (MMR^−^ or WT), the sum of G scores was computed for measuring the extent of parallel evolution. The arrow and vertical dashed line show the observed sum of G scores for each treatment combination. The histogram represents the null distribution of evolutionary parallelism as determined by 20,000 simulated sums of G scores, where the positions of mutations were randomized throughout the genome. The significance of the observed sums is evaluated by *z* scores, which quantify the extent the observed value deviates from the null distribution (*z* > 1.96 for two-tailed *P* < 0.05). (B) Similarly, the arrow and vertical dashed line show the observed mean of Bray-Curtis similarity across the pairs of evolved populations evolved in a combination. The histogram represents the null distribution of evolutionary parallelism as determined by 1,000 simulated means of Bray-Curtis similarity, where the positions of mutations were randomized throughout the genome. The significance of the observed sums is evaluated by *z* scores (*z* > 1.96 for two-tailed *P* < 0.05). Download FIG S1, PDF file, 0.3 MB. Copyright © 2022 Behringer et al. 2022 Behringer et al. https://creativecommons.org/licenses/by/4.0/ This content is distributed under the terms of the Creative Commons Attribution 4.0 International license.

Gene ontology (GO) analysis of genes that were determined to significantly contribute to the increased sum of *G* scores revealed that genes associated with DNA repair (GO: 0006281) or replication (GO: 0006260) more commonly experienced mutations in 10-day populations than in 1-day populations (Table S1). Further, genes associated with fatty acid metabolism (*fabF*, *plsX*, and *putP*), nutrient uptake (*ompF*, *ompR*, *oppA*, *oppC*, and *sstT*), and metal scavenging (*fur* and *pitA*) were also observed as common targets of mutation (Fig. 2B and C and Table S2A and B). As fatty acid biosynthesis (46) and metal scavenging (47) are metabolically costly processes, alterations of these functions may be beneficial under energy-limited conditions, such as those experienced 10 days posttransfer into LB broth.

10.1128/mbio.03467-21.4 TABLE S1 Mutations in DNA replication and repair genes. Download Table S1, XLSX file, 0.02 MB. Copyright © 2022 Behringer et al. 2022 Behringer et al. https://creativecommons.org/licenses/by/4.0/ This content is distributed under the terms of the Creative Commons Attribution 4.0 International license.

10.1128/mbio.03467-21.5 TABLE S2 G scores of genes significantly enriched for nonsynonymous mutations (A) or structural mutations (B) across evolved populations. Download Table S2, XLSX file, 0.01 MB. Copyright © 2022 Behringer et al. 2022 Behringer et al. https://creativecommons.org/licenses/by/4.0/ This content is distributed under the terms of the Creative Commons Attribution 4.0 International license.

### Sequencing of clones from a 10-day population confirms distinct ecotypes.

**FIG 3:**
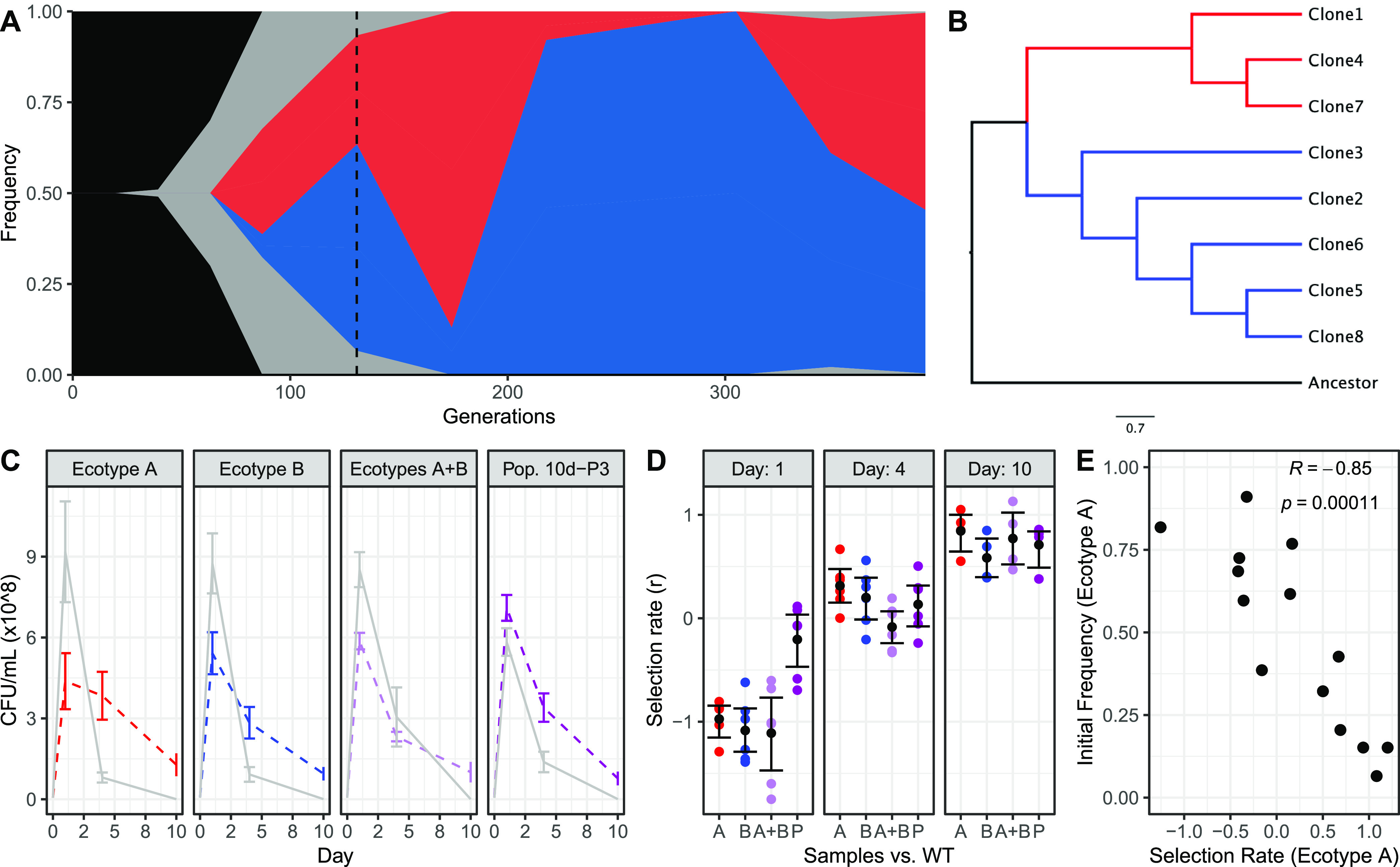
Evaluation of isolated clones reveals long-term ecotype dynamics suggesting frequency-dependent selection. (A) Pairing whole-population genomic sequencing of population 10d-P3 (WT, 10-day cycles) with genomic sequencing of eight clones isolated from population 10d-P3 at the day 300 time point (dotted vertical line) reveals two distinct ecotypes, ecotype A (red) and ecotype B (blue). (B) Clustering via maximum likelihood confirms the distinct diversification of isolated clones into two primary clades. (C) Coculture assays competing the WT ancestor against isolated clones (A, red; B, blue), pooled clones (1:1 A and B, light purple), and revived population samples (1:2 A and B) over 10 days of culture suggests frequency-dependent cooperative interactions between ecotypes, as evolved samples perform worse outside the evolved 1:2 ratio observed at the day 300 time point. Lines denote the trend of the mean CFU/mL count of the ancestor (solid gray) and evolved (dashed colored) samples over the 10-day coculture. Error bars denote ± SEM. (D) Revived population samples (P) exhibit significantly higher selection rates on day 1 compared to the other evolved samples, with comparable selection rates on day 4 and 10. Colored circles indicate measured selection rates for each replicate, black circles indicate mean selection rate, and error bars indicate ± SEM. (E) Competition of ecotype A versus ecotype B when pooled at different initial frequencies reveals selection rate values that confirm negative frequency dependence. R value denotes Pearson’s correlation coefficient.

Sampling and sequencing clones from evolved 10-day populations can help identify individuals that belong to each ecotype and further resolve the extent of diversification (35). This approach consists of isolating clones from an evolving population at an intermediate time point and associating identified mutations from these clones with the mutation frequencies in the time-series metagenomic sequencing data. Thus, to further understand the population dynamics that evolved in response to 10-day feast/famine cycles, we focused on eight clones randomly isolated from a single population, population 403 (here referred to as population 10d-P3), at the day 300 time point. This population was selected because it had an initially WT genetic background and experienced one of the less extreme increases in basal mutation rate, thus rendering a smaller number of total mutations and an increased ability to connect genotypes to phenotypes. Across all eight clones from population 10d-P3, we identified 82 single nucleotide polymorphisms (SNPs), 16 small indels, 14 IS element insertions, and 5 large deletions, for an average of 43.12 ± 5.3 mutational events per clone (Table S3). Comparison of the identified mutations from clone sequences to the time-series metagenomic data suggests the presence of two major clades. Here, shared mutations in ecotype A (clones 1, 4, and 7) accounted for ~35% of the population and ecotype B (clones 2, 3, 5, 6, and 8) accounted for ~65% of the population based on SNP frequency at 300 days (Fig. 3A). We further confirmed these two major clades using an approximated maximum likelihood method for inferring relatedness by SNP alignments (Fig. 3B) (48). Among the mutations identified in sequenced clones, there were 23 mutations that had arisen across 18 of the genes identified as targets of parallel evolution under 10-day feast/famine cycle conditions (Fig. 2B and C and Table S3). Seven of these 18 genes had mutations that were present in all of the isolated clones, while mutations in the other 11 genes were specific to one of the two observed ecotypes that evolved in population 10d-P3. Mutations specific to ecotype A were located in genes associated with amino acid degradation (*dadA*, *mhpR*, *putA*, and *putP*) or fatty acid biosynthesis (*plsX*), while ecotype B had mutations located in genes associated with fatty acid elongation (*fabF*) and resource import (*ompF*, *ompR*, *oppA*, and *sstT*).

10.1128/mbio.03467-21.6 TABLE S3 Mutations identified in population 10d-P3 clones at the day 300 time point. Download Table S3, XLSX file, 0.02 MB. Copyright © 2022 Behringer et al. 2022 Behringer et al. https://creativecommons.org/licenses/by/4.0/ This content is distributed under the terms of the Creative Commons Attribution 4.0 International license.

To assess how this ecotypic diversification impacted the evolution of fitness in evolved clones, we performed competitive fitness assays across a single 10-day feast/famine cycle and then counted CFU on days 1, 4, and 10. Here, we cocultured the WT ancestor with a representative clone from each ecotype, a 1:1 pooled sample of both ecotypes, or the revived 10d-P3 population at the day 300 time point. Clone 1 was selected to represent ecotype A; clone 2 was selected to represent ecotype B. The fitness advantage of the evolved clone(s) or population compared to the ancestor was calculated by the difference between their Malthusian parameters, which represents the selection rate, [ln(Evolved_CFU Final_/Evolved_CFU Initial_)/days] – [ln(WT_CFU Final_/WT_CFU Initial_)/days] (49) (Fig. 3C and D). In response to coculture, both representative clones were less competitive than the WT ancestor by day 1 but could outcompete the WT ancestor by day 4, consistent with patterns indicating a trade-off between growth and longevity (50). Interestingly, when fitness was assessed in the context of within-population interactions, a pattern indicative of potential frequency dependence emerged. When cocultured with the WT ancestor, pooled samples of both ecotypes were only found at higher abundances than the WT ancestor on day 10, but the revived population sample was found on all days to be at equal or greater abundances than the WT ancestor. The advantage of the revived population sample is best observed on day 1, where the population exhibits the highest selection rate compared to the representative clones or the 1:1 pooled clones (*P*_PvsA_ = 0.009; *P*_PvsB_ = 0.002; *P*_PvsA+B_ = 0.001; ANOVA with Tukey’s HSD) (Fig. 3D). However, on day 4 and day 10 of coculture, there is no significant difference among the selection rates of the representative clone, the 1:1 pooled clones, and the revived population. Competition of ecotype A against ecotype B by pooling the clones at different initial frequencies confirmed negative frequency-dependent selection, as both ecotype A and ecotype B exhibit their highest selection rates when they are rare (Pearson’s *R* = −0.85; *P = *0.00011).

### Transcriptomes reveal ecotype-specific differences in gene expression.

Because evolution to 10-day feast/famine cycles appears to manifest additive effects among clones as well as a trade-off between replicative fitness and longevity, we characterized how gene expression evolved at both the population and the ecotype levels of population 10d-P3. To examine common gene expression changes that evolved as a response to 10-day feast/famine cycles and identify differentially expressed genes (DEGs) that may contribute to the growth/longevity trade-off, we focused on the expression changes during exponential growth and late stationary phase. Specifically, RNA was collected at the relative mid-log time point and at 36 h for both of our representative clones and the WT ancestor (Fig. S2). When comparing the ecotypes together as a group against the WT ancestor, we found a significantly greater number of DEGs at mid-log phase (*n* = 994, 23.9% of genes) than at 36 h (*n* = 406, 9.7% of genes; *P < *2.2 × 10^−16^, *z* test for proportions) (Table S4). For both time points, these changes in gene expression were biased toward reduced expression (*P*_mid-log_ = 1.84 × 10^−13^; *P*_36h_ = 1.07 × 10^−10^, binomial test). To gain insight on the processes that were targets of differential expression, we looked for the significantly enriched GO terms across DEGs (Table S5). At mid-log phase, 10-day clones exhibit increased expression of translation machinery and iron uptake/homeostasis genes and decreased expression of stress response and anaerobic metabolism genes. Alternatively, at 36 h, genes associated with phenylacetate catabolism, IMP biosynthesis, and thiamine diphosphate biosynthesis exhibit increased expression, while no significant enrichment of GO terms was found for genes that exhibit decreased expression.

10.1128/mbio.03467-21.2 FIG S2 Quenching of cell growth for transcriptomic analysis is based on the growth and metabolic activity of ancestor and ecotype clones. (A) Growth curves were assessed in a 96-well plate reader to determine relative mid-log growth for the WT ancestor (black) and representative clones for ecotype A (red) and ecotype B (blue). Solid filled circles represent the mean trend of growth for each clone type, and error bars represent ± SEM for each time point (15-min intervals). Vertical dashed lines highlight the relative mid-log phase for each clone type (ancestor, 4 h; ecotype A, 5.75 h; ecotype B, 5.75 h). (B) Flow cytometry quantifying the fraction of ancestor cells in live (circles), dead (squares), or compromised (diamond) states was used to determine the sampling time for late stationary phase. Solid, filled shapes represent the mean cell fraction after 24 h, 48 h, and 96 h; error bars represent ± SEM for each time point. We ultimately selected 36 h, as >90% of ancestor cells were still present in an active metabolic state at this time point. Download FIG S2, PDF file, 0.04 MB. Copyright © 2022 Behringer et al. 2022 Behringer et al. https://creativecommons.org/licenses/by/4.0/ This content is distributed under the terms of the Creative Commons Attribution 4.0 International license.

10.1128/mbio.03467-21.7 TABLE S4 Differentially expressed transcripts in population 10d-P3 at mid-log phase and 36 h. Download Table S4, XLSX file, 0.2 MB. Copyright © 2022 Behringer et al. 2022 Behringer et al. https://creativecommons.org/licenses/by/4.0/ This content is distributed under the terms of the Creative Commons Attribution 4.0 International license.

10.1128/mbio.03467-21.8 TABLE S5 GO terms associated with differentially expressed transcripts in population 10d-P3. Download Table S5, XLSX file, 0.02 MB. Copyright © 2022 Behringer et al. 2022 Behringer et al. https://creativecommons.org/licenses/by/4.0/ This content is distributed under the terms of the Creative Commons Attribution 4.0 International license.

**FIG 4:**
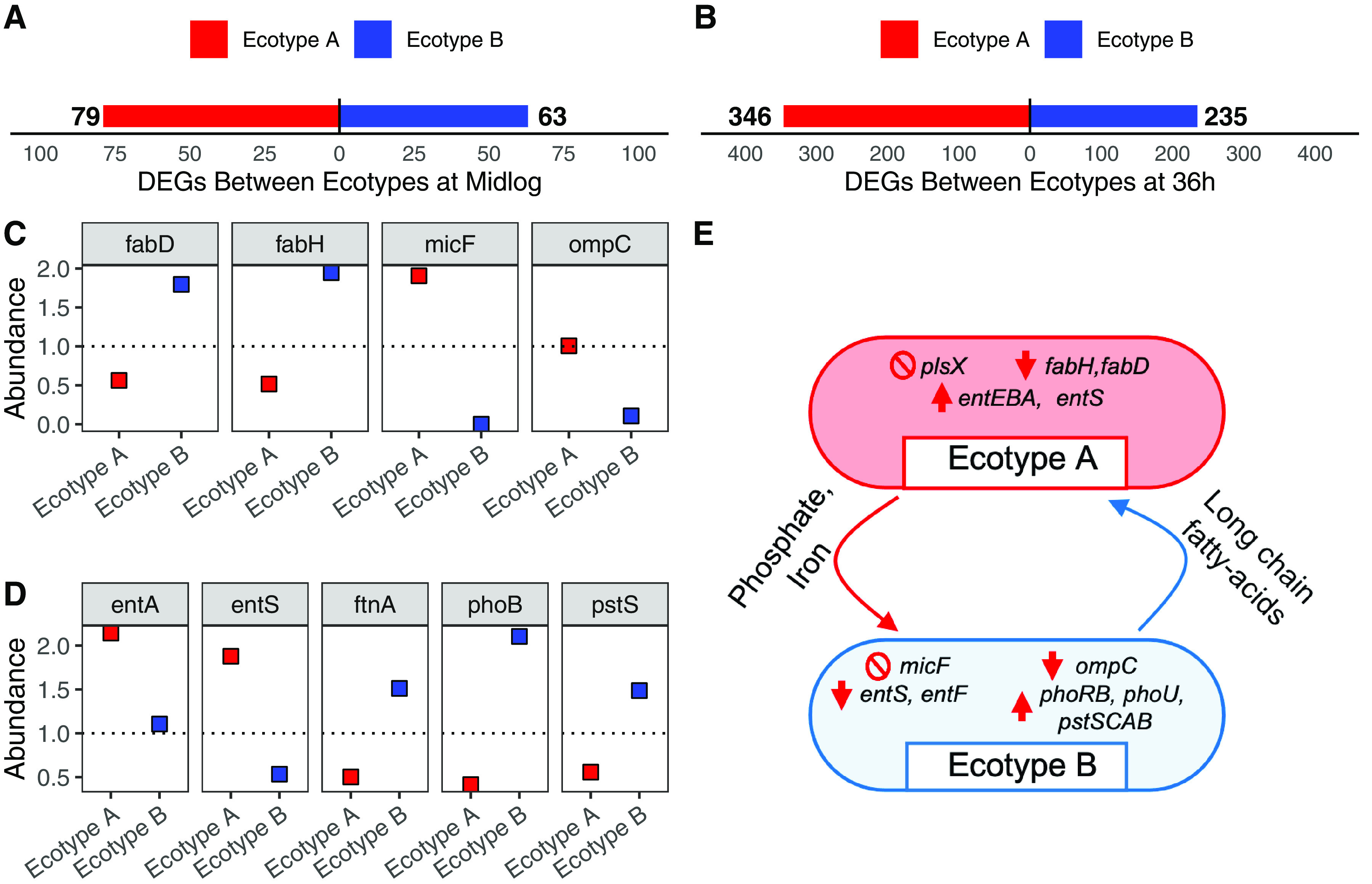
Differential expression reveals functional differences between evolved ecotypes. Comparison of relative transcript abundance between ecotypes at mid-log phase (A) and 36 h (B). Bars illustrate the total number of transcripts with significantly higher expression in ecotype A (red) or ecotype B (blue). (C) Relative abundance of transcripts associated with fatty acid biosynthesis (*fadD* and *fadH*) and passive uptake of long-chain fatty acids (*micF* and *ompC*) for both ecotypes with respect to mean transcript abundance of the WT ancestor at mid-log phase. (D) Relative abundance of transcripts associated with enterobactin biosynthesis (*entA* and *entS*), iron storage (*ftnA*), and phosphate uptake (*phoB* and *pstS*) for both ecotypes with respect to mean transcript abundance of the WT ancestor at 36 h. For both panels C and D, ecotypes A and B are represented by red and blue squares, respectively. The dotted line represents the 1:1 ratio line with respect to the WT ancestor. (E) Schematic illustrating the hypothesized cross-feeding relationships between ecotypes A and B, with crossed circles highlighting null genes of interest, thick red arrows illustrating evolved differences in gene expression, and thin curved arrows illustrating resources shared by ecotype A to ecotype B (red) or shared by ecotype B to ecotype A (blue).

Next, to investigate potential sources of biological interaction that could contribute to the increased fitness observed in the evolved population, we compared the transcriptomes of the ecotypes to each other and screened for DEGs that indicate cooperative behaviors or complementary roles within the population. A gene was considered differentially expressed between ecotypes if its log_2_ ratio of transcript counts (${\text{log}}_{2}\frac{\text{Ecotype A}}{\text{Ecotype B}}$) was >1.5 (higher expression in ecotype A) or <−1.5 (higher expression in ecotype B). To determine if the source of differential expression between ecotypes was due to an evolved increase or decrease of expression in a particular ecotype, transcript counts of DEGs were compared to the mean normalized transcript count of the WT ancestor replicates. In contrast to the population changes, the number of DEGs between ecotypes is lower at mid-log phase and greater at 36 h, suggesting specific adaptive diversification between ecotypes for enhanced survival during long-term stationary phase (Fig. 4A and B and Table S6). Of the DEGs between ecotypes identified at mid-log phase, a pattern also emerged to suggest that cross-feeding on fatty acids evolved (Fig. 4C). Specifically, ecotype A evolved reduced expression of *fabD* and *fabH*, while ecotype B evolved increased expression of these genes. Both *fabD* and *fabH* are annotated as essential for growth in LB broth (51) and encode the enzymes responsible for the initiation of fatty acid biosynthesis. Reduced expression of *fabD* and *fabH* in ecotype A is likely due to polar effects from an IS*186* insertion in *plsX*, located in the same operon. In addition, ecotype B evolved a large deletion that involved the small RNA *micF* and a large portion of the operator region of *ompC*, resulting in a significant decrease of *ompC* expression. Deletion of *ompC* has been shown to result in a significant decrease in fatty acid uptake and reduced competition for free fatty acids in the media (52). Alternatively, at 36 h, DEGs between ecotypes revealed another candidate for cooperation based on iron uptake. Ecotype A evolved increased expression of genes normally repressed by the iron uptake regulator, Fur-Fe^2+^, including siderophore biosynthesis (*entA*, *entB*, and *entE*) and export (*entS*) (Fig. 4D). In return, ecotype B evolved decreased expression of many of these genes and increased expression of the ferritin iron storage complex (*ftnA*), which is induced by Fur-Fe^2+^. In addition to iron ion homeostasis, phosphate starvation also emerged as a candidate for cooperation at 36 h based on DEGs between ecotypes. Here, ecotype B evolved increased expression of genes associated with the phosphate two-component regulation system (*phoB*, *phoR*, and *phoU*) and phosphate ABC transport (*pstS*, *pstC*, *pstA*, and *pstB*) (Fig. 4D). These specialized roles between ecotypes may have repeatedly evolved in our 16 evolving populations in response to the 10-day feast/famine cycles, given the similarity in the DEGs between ecotypes and the genes identified as highly parallel mutational targets in 10-day populations. Further investigation is needed to confirm these cross-feeding relationships, the order in which they evolved, and how each component contributes to population-level fitness.

10.1128/mbio.03467-21.9 TABLE S6 Differentially expressed transcripts between ecotypes of population 10d-P3. Download Table S6, XLSX file, 0.09 MB. Copyright © 2022 Behringer et al. 2022 Behringer et al. https://creativecommons.org/licenses/by/4.0/ This content is distributed under the terms of the Creative Commons Attribution 4.0 International license.

## DISCUSSION

Through this study, we found that ecotypic diversification repeatedly evolves in Escherichia coli populations cultivated in 10-day feast/famine cycles. While long coexistence of diversified clades is a phenomenon that was also previously observed in populations cultivated in 1-day feast/famine cycles, 10-day-cycle populations additionally evolve to produce even thicker biofilms and harbor higher base genetic mutation rates than both the ancestral strains and the 1-day cycle populations. Together, the increases in evolved trait values and the genomic evidence reveal a stronger signal of mutational parallelism under 10-day feast/famine conditions. The 10-day feast/famine conditions provide a different and potentially more challenging environment for evolving E. coli populations than the 1-day feast/famine conditions, which might account for the increased mutational parallelism despite experiencing 1/10th fewer generations. It is important to note that the starvation conditions of 10-day feast/famine cycles also induce differences in population-size dynamics and mutation rate compared to 1-day conditions. Therefore, the effect of starvation, population size change, rate of evolution, or any combination of these might explain the unique evolutionary outcomes in 10-day cycles. Further investigation of clones isolated from a 10-day population illustrates that ecotypic diversification is rapid, results in individual fitness trade-offs with additive effects, and is based on differential investment in the biosynthesis or acquisition of key resources, such as fatty acids, iron, and phosphate. These individual fitness trade-offs are distinct to 10-day feast/famine cycles, as isolated clones from 1-day cycle populations all exhibited fitness increases in their evolved environment independent of being cocultured with their evolved community members (35). Thus, the stress invoked due to extended resource limitation experienced in 10-day cycles appears to encourage the evolution of bidirectional cost-sharing for metabolically expensive processes.

**FIG 5:**
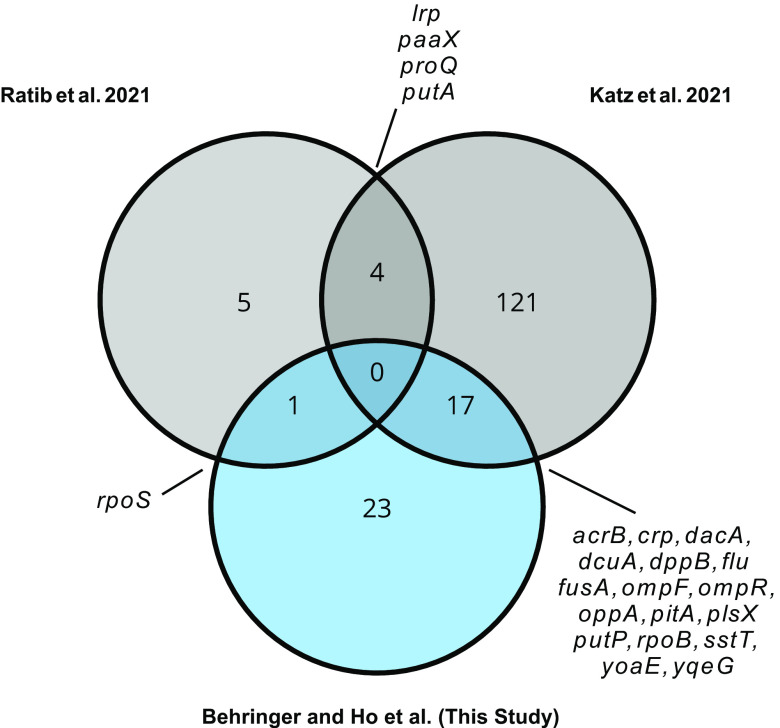
Overlapping targets of parallel mutation following evolution of E. coli populations to long-term starvation and 10-day feast/famine cycles. Venn diagram showing the number of overlapping genes experiencing parallel mutations as a result of evolution to 10-day feast/famine cycles (this study; blue) and long-term starvation (Ratib et al. [57] and Katz et al., 2021(30); gray). Circles representing each gene set are not drawn to scale, and genes that overlap between multiple studies are listed in the offset labels.

Prior to this study, there were few investigations into how adaptation to resource limitation is shaped by feast/famine cycles (53, 54). Instead, interpretations of how E. coli adapts to increasingly diminishing resources have been drawn primarily from studies focused exclusively on the famine component (55, 56), such as investigations of the growth advantage in stationary phase (GASP) phenotype. However, in analysis of how the eventual feast component factors into adaptation, one can only observe mutations that allow E. coli to postpone death during starvation and famine conditions. As resources must eventually be replenished in nature or the population will ultimately go extinct, it is important to examine adaptation to starvation in the context of subsequent resource replenishment, which helps identify the starvation-associated traits and alleles that are beneficial when resources are scarce but are not overly detrimental when resources are abundant. A comparison of the genes that experienced parallel mutations during 10-day feast/famine cycles (Fig. 2B and C) to the genes reported as targets of parallel mutation in previous studies of long-term starvation (30, 57) revealed 22 genes that overlap between two or more studies (Fig. 5). Of these overlapping genes, four (*lrp*, *paaX*, *proQ*, and *putA*) were exclusively observed as targets of parallel mutation in studies of long-term starvation. Thus, mutations in these genes may be deleterious upon resource replenishment or may only confer benefits during deep starvation. Alternatively, 18 genes were targets of parallel mutation in both 10-day feast/famine cycle and under long-term starvation conditions, including global regulators and genes with broad effects on transcription and protein expression (i.e., *crp*, *fusA*, *ompR*, *rpoS*, and *rpoB*). The continued presence and eventual fixation of these mutations following multiple 10-day resource replenishment cycles suggests that these mutations have a net benefit over the complete replenishment cycle.

In addition to parallel mutations, we observed evolved changes affecting multiple traits that have been associated with adaptation to starvation in prior studies. One such trait is a trade-off between growth and survival under starvation conditions (30, 58). When determining the time at which 10-day clones and the WT ancestor reach mid-log phase to ensure that our differential expression analysis would capture physiologically relevant comparisons, we observed that the midpoint of logarithmic growth occurred 105 min later for the evolved 10-day-cycle clones (see Fig. S2 in the supplemental material). Despite this more languid growth, both evolved 10-day-cycle clones exhibited increased starvation tolerance in coculture assays and outnumbered the WT ancestor by 4 days posttransfer (Fig. 3C and D). Recent study of E. coli growth and survival in batch culture compared to chemostat culture, where growth rates can be controlled, revealed that lower growth rates translated to lower death rates, as these cells required fewer resources per unit of time to maintain viability (50). Moreover, in addition to evolving lower growth rates, 10-day clones evolved changes in gene expression that were largely biased toward reduced expression and presumably result in lower cellular maintenance costs.

A second trait that exhibited significant differences following evolution to feast/famine cycles was the base genetic mutation rate. Here, we observed that increased mutation rates occurred much more frequently in populations under 10-day cycles, a presumably more stressful environment, than 1-day cycles. Further, as most initially WT populations eventually evolved defects in MMR during evolution to 10-day feast/famine cycles, we observed few differences between initially WT and initially MMR^−^ populations. Consistent with our results, the emergence of high mutation rates has been found in many other microbial evolution experiments in response to various conditions (29, 59, 60). Population-genetic models have also suggested that higher mutation rates can evolve in fluctuating environments by hitchhiking with other beneficial mutations (61, 62). Mutators are also expected to be at their most prevalent when environmental fluctuations occur with intermediate rates of change, in contrast to rapid fluctuations where the benefit of a mutator is lost when environmental changes occur faster than the beneficial mutation rate (63). Under all conditions, however, as a population becomes better adapted to its environment, the pool of available beneficial mutations shrinks. At this point, the fitness costs associated with high mutation due to an increased fraction of deleterious mutations can outweigh the benefits of a rare beneficial mutation, ultimately leading to natural selection favoring compensatory evolution and reversion to lower mutation rates (64). Determining whether that will be also the case in the evolved 10-day feast/famine populations will require future follow-up as these populations continue to evolve under these fluctuating resource conditions.

Other traits that are being increasingly associated with microbial adaptation to resource limitation are diversification and spatial organization. In response to 10-day feast/famine cycles, evolving populations exhibited rapid diversification and increased biofilm formation activity. This result echoes what has been observed in other studies, in which various degrees of resource limitation exhibited positive effects on spatial structure and diversity. Studies of patterning in bacterial colonies have revealed that spatial organization is a common outcome of resource limitation due to bottlenecks during colony expansion, and the speed at which bacteria spatially organize increases with resource limitation (65). Under heterogeneous culture conditions, unimodal relationships between maintenance of diversity and resource availability have been described, with peak diversity ultimately observed in intermediate nutrient concentrations (66). Further similarities between our E. coli system and these two studies include the utilization of an experimental ancestor strain that expresses poor motility (67), which was observed to increase spatial patterning in Pseudomonas aeruginosa (65), and biofilm formation at the surface-air interface, which underlies the diversity observed in the heterogeneous Pseudomonas fluorescens system (66). Further study is needed to parse the importance of reduced motility and increased biofilm formation to the generation and maintenance of diversity across resource-limited conditions.

As a result of diversification, evidence of frequency-dependent cooperative behavior was observed between clones isolated from evolved populations. Here, isolated clones exhibited growth disadvantages when independently cocultured with the WT ancestor and when pooled at a 1:1 ratio. However, these growth disadvantages were minimized when a revived sample of the population was cocultured with the WT ancestor, with the ecotypes competing at a more favorable 1:2 ratio, and competition of the ecotypes against each other shows that the individual ecotypes exhibit increased selection rates when rare. As both ecotypes are viable in isolation, they have not evolved obligate dependence, and any evolved changes contributing to this cooperative behavior are likely to increase the metabolic efficiency of the population but at a cost to individual fitness. Mediating these costs to individual fitness has proven challenging to evolutionary biology. However, a relatively recent hypothesis, called the black queen hypothesis, has emerged as a leading theory describing the evolution of cooperation by genomic reduction or gene loss (19). Here, mutualism can arise based on overproduction of metabolic intermediates that leak from the cell, providing a public good. In response, other community members may lose the ability to biosynthesize (or reduce their investment in the biosynthesis of) the now freely available public good. If this exchange is bidirectional, then codependence may evolve. One major caveat to the black queen hypothesis is an innate vulnerability of mutualistic cross-feeding relationships to invasion by cheaters that benefit from the public goods without contributing (68). As such, mutualistic cross-feeding is expected to be (i) the most stable in structured environments (69), where access to the public goods can be limited; (ii) subject to Allee effects (70), where an optimal ratio of each cooperator type exists, maximizing metabolic efficiency and population size while also reducing an excess availability of public goods that may otherwise encourage the evolution of cheaters; and (iii) beneficial under resource-limited conditions (15), where cooperation can help maintain genetic variation. All of these conditions appear to be true for our experimental populations, as 10-day feast/famine cycles result in increased biofilm formation and an optimum 1:2 ratio exists between ecotypes for maximum population fitness.

Lastly, the metabolic basis of cross-feeding observed between evolved ecotypes is likely parallel across our experimental populations, representing a common evolutionary outcome of culture under 10-day feast/famine cycle conditions. Differential expression analysis revealed that ecotype A evolved significantly reduced expression of essential fatty acid biosynthesis genes, *fabD* and *fabH*, likely due to the polar effect of an IS element insertion in the upstream gene, *plsX*. Similarly, *fabH* and *plsX* were common targets of mutations in our other 10-day populations, with *fabH* mutations arising in eight populations and *plsX* mutations arising in six populations (Fig. 2 and Table S7). In a complementary fashion, ecotype B evolved significantly reduced expression of enterobactin exporter *entS* and other genes regulated by Fur-Fe^2+^ during long-term stationary phase. Across the other 10-day populations, *fur* was also a common target of mutation (10 populations), with three populations containing nonsynonymous mutations resulting in substitutions of the R110 residue, which is proximal to Glu108 of Fur’s metal binding site 1 (71). The potential involvement of long-chain fatty acids and iron sequestration in cross-feeding mutualisms is significant, as both of these processes are energetically costly, specifically when considering that synthesizing palmitate (C_16:0_), the precursor to other long-chain fatty acids, costs 8 acetyl-coenzyme A (CoA), 14 NADPH, and 7 ATP molecules (46). Thus, the evolution of cross-feeding to minimize the investment in palmitate production is consistent with the presumption of the black queen hypothesis that cross-feeding will evolve to conserve energy wasted by redundant synthesis of costly metabolites. Further study is needed to dissect the metabolic interactions between these evolved ecotypes as well as the stepwise process that leads to these cooperative interactions and their long-term evolutionary fate within populations.

10.1128/mbio.03467-21.10 TABLE S7 Mutations observed in *plsX* and *fabH* across evolved populations. Download Table S7, XLSX file, 0.01 MB. Copyright © 2022 Behringer et al. 2022 Behringer et al. https://creativecommons.org/licenses/by/4.0/ This content is distributed under the terms of the Creative Commons Attribution 4.0 International license.

### Conclusions.

Our results illustrate how long-term experimental evolution to cycles of feast and famine encourages rapid ecotypic diversification. Further analysis of a representative population reveals that evolved ecotypes may participate in cooperative behavior based on bidirectional cross-feeding, reminiscent of the black queen hypothesis. Here, ecotypes evolved mutations targeting costly processes such as fatty acid biosynthesis and iron sequestration, representing a substantial energy savings if these processes can be supplemented by environmental sources or via other microbial community members. Given that cooperative bidirectional cross-feeding is often observed in nature and that the investigation of how cooperative behaviors evolve has largely relied on engineered relationships (69, 72–74), these populations evolved under 10-day feast/famine conditions represent a great resource for the study of how cooperative behaviors evolve *de novo*.

## MATERIALS AND METHODS

### Strains and experimentally evolved populations.

The experimentally evolved populations originating with WT genetic backgrounds are descendants of PFM2, a prototrophic derivative of E. coli K-12 strain MG1655 (MG1655, *rph*^+^). The experimentally evolved populations originating with MMR^−^ genetic backgrounds are descendants of PFM5 (PFM2, Δ*mutL*). PFM2 and PFM5 were gifted by the Foster lab (34). For half of the WT and MMR^−^ evolved populations, the *araBAD* operon in the starting clone was further disrupted via P1 transduction (PMF2/PMF5, Δ*araBAD567*).

Experimental evolution was started by the inoculation in 10 mL of LB-Miller broth in a 16- by 100-mm culture tube and then maintained in a shaking incubator at 175 rpm at 37°C. Every day or every 10 days, the liquid culture was vortexed, and 1 mL of the vortexed liquid culture was transferred to another tube with fresh LB broth for a 1:10 dilution. By using a 1:10 dilution we maximize effective population size while still allowing enough generations to occur and ensuring suitable introduction of genetic variation for each transfer. To maintain a rich historical record of evolution, 30 days or every 100 days we collected 1 mL of pretransfer culture, which was subsequently frozen in 40% glycerol and stored at −80°C. Additional 1-mL aliquots of pretransfer culture that were collected for DNA sequencing were centrifuged to remove the spent media, frozen with liquid nitrogen, and stored at −80°C. On days 90, 200, 300, 400, 500, 600, 700, 800, and 900, we screened for contaminated populations by MacConkey agar (BD Difco) with arabinose (0.4%).

To further investigate a representative population and the evolved ecotypic diversification, eight clones were arbitrarily selected after plating a revived overnight culture of population 403 (10d-P3) from the day 300 time point. Isolated clones were restreaked on LB agar, incubated overnight, transferred into cryovials containing 40% glycerol, and stored at −80°C.

### Quantification of biofilm formation.

To quantify evolved differences in the ability to form biofilms, we used a microtiter plate biofilm assay (75). In a non-tissue-treated 96-well plate (351172; Corning/Falcon), 15 μL of overnight culture was inoculated into 150 μL of LB broth. Each assessed population was measured for a total of eight replicates, and the *ΔaraBAD ΔmutL* ancestor was measured for a total of 16 replicates. As a control, each 96-well plate contained two columns (16 wells) of the *ΔaraBAD* WT ancestor and at least two columns (16 wells) of blanks. Inoculated 96-well plates were incubated for 24 h at 37°C. Following incubation, plates were washed with 1× phosphate-buffered saline (PBS), stained with 0.1% crystal violet for 10 min (200 μL per well), washed again with 1× PBS, and left to dry overnight. Crystal violet bound to biofilms was then solubilized with 30% acetate for 15 min (200 μL per well) and transferred to a fresh 96-well plate, and absorbance was quantified using an Epoch2 microplate spectrophotometer at 550 nm (WT, *n*_ans_ = 192, *n*_1-day_ = 48, *n*_10-day_ = 32; MMR, *n*_ans_ = 16, *n*_1-day_ = 48, *n*_10-day_ = 32).

### DNA extraction and sequencing of clones and populations.

On days 90, 200, 300, 400, 500, 600, 700, 800, and 900, 1 mL of each experimentally evolved population was collected for DNA extraction. We used a DNeasy UltraClean microbial kit (Qiagen 12224; formerly MO BIO UltraClean Microbial DNA kit) for extraction. Library preparation and sequencing were performed either at the Hubbard Center at the University of New Hampshire (UNH), the Center for Genomics and Bioinformatics at Indiana University, or the CLAS Genomics Facility at Arizona State University (ASU) for library preparation and sequencing. During library preparation, the Nextera DNA library preparation kit (FC-121-1030; Illumina) was first used, and an augmented protocol for optimization of reagent use (76) was then performed. During the sequencing, we used paired-end reads on an Illumina HiSeq 2500 (UNH) or an Illumina NextSeq 500 (Indiana, ASU) with a target depth of 100×. DNA sequencing of isolated clones followed an identical protocol except that clones were revived from frozen storage on LB agar by streaking for isolation and incubated overnight at 37°C. The following day, a single isolated colony was selected for DNA extraction.

### Sequencing processing and mutation calling.

Population and clonal sequencing reads were preprocessed using Cutadapt v.1.9.1 (77) to remove residual adapters and trim low-quality sequences. After this quality control step, we mapped population-level metagenomic sequencing reads to the reference genome of Escherichia coli K-12 substrain MG1655 (NC_000913.3) with the Breseq v.0.30.2 pipeline, which also called mutations and their frequencies with the predict-polymorphisms parameter setting (78). In addition, we only focused on the samples that passed the following four criteria: (i) mean sequencing depth of >10; (ii) proportion of genome with zero depth of <5%; (iii) not identifying the 1,83-bp deletion in *mutL* for a WT population; and (iv) identifying the correct set of mutations in terms of *ΔaraBAD* marker (including a nonsynonymous SNP at position 66528, an intergenic SNP at position 70289, and a multiple-base substitution mutation [SUB] at position 66533). An *ara*^+^ line was kept in the analysis if we found either of two SNPs with a derived allele frequency (DAF) of <0.2. An ΔAraBAD line was kept in the analysis if we found either of two SNPs with DAF of >0.8 or the SUB is called. In the end, 275 genomic profiles were used in the analysis (data available on GitHub at https://github.com/LynchLab/ECEE_Starvation). If the mutations were known to exist in the ancestral lines, they were removed from the analysis. We also removed the mutations with a DAF of 100% at one time point for at least 11 experimental populations with the same genetic background from the analysis. In addition, we also remove mutations associated with *rsx* genes, as the sequences are highly repetitive and are known to cause errors in SNP calling (79). For clonal sequences, we mapped genomic sequencing reads to the E. coli reference genome by following GATK best practices (https://gatk.broadinstitute.org/).

### caHMM.

We performed a clade-aware hidden Markov model (caHMM) using a modified version of previously released code (9) to test coexistence time in each experimental population. We used the following modifications: (i) continuing the iteration of populations even when the major-clade frequency of all time points cannot be estimated; (ii) increasing the iteration number for hidden Markov chain from 5 to 50; and (iii) removing the original cutoff for minimum coexistence time, as that parameter is too long for our experiments. For caHMM analysis, we constructed an annotated time course of mutations using the original format. The minimal generation number of 3.32 between two transfers was assumed, as the dilution factor is 1:10. The sequencing depth of a structural mutation (DEL, INS, and MOB) is determined by the mean sequencing depth across the mapped adjacent nucleotide (“coverage_plus” or “coverage_minus”) of associated junction candidates (JCs) in the Breseq annotated file. The sequencing depth of time zero was set to 100. The calculation of the longest coexistence time was based on the major-clade frequency (*f*_M_) and minor-clade frequency (*f*_m_) inferred by caHMM analysis. Specifically, if no time points show 0.2 < *f*_M_ <0.8 and 0.2 < *f*_m_ <0.8, the longest length of coexistence is set to zero. Otherwise, we found the longest time intervals where we included every time point show 0.01 < *f*_M_ <0.99 or 0.01 < *f*_m_ <0.99. If the caHMM analysis could not finish for an experimental population, we instead performed the well-mixed hidden Markov chain (wmHMM) and made the longest length of coexistence zero. The single clade in wmHMM is defined as the basal clade. Candidate mutations for adaptation are annotated as fixed mutations in the basal, major, or minor clade in the caHMM/wmHMM output or showing DAF of >0.5 for at least two time points.

### Calculation of *G* scores.

For each resource-replenishment cycle/genetic background combination, we calculated the *G* score of candidate nonsynonymous mutations for each gene (43) to quantify the parallelism of the set of candidate nonsynonymous mutations. A larger *G* score means more overrepresentation for a gene. Specifically, for each resource-replenishment cycle/genetic background combination, we counted the observed number of nonsynonymous mutations in gene *i* (*O_i_*). We then estimated the expected number of nonsynonymous mutations in gene *i* (*E_i_*) by *O_tot_*(*L_i_*/*L_tot_*), where *O_tot_* = Σ*_i_O_i_*, *L_i_* is the number of nonsynonymous sites for gene *i*, and *L_tot_* = Σ*_i_L_i_*. In the end, the *G* score for gene *i* (*G_i_*) was quantified by 2*O_i_*ln(*O_i_*/*E_i_*), or *G_i_* = 0 when *O_i_* = 0. Note that when 0 < *O_i_* < *E_i_*, 2*O_i_*iln(*O_i_*/*E_i_*) < 0, which leads to a paradoxical scenario that *G_i_* = 2*O_i_*iln(*O_i_*/*E_i_*) for a gene with a few mutations can be smaller than *G_i_* = 0 for the same gene with no mutations. Therefore, we also defined *G_i_* = 0 when 2*O_i_*ln(*O_i_*/*E_i_*) < 0.

We noted that the null expectation of *G* scores is different when *O_tot_* is different (see Fig. S3 in the supplemental material). Therefore, 20,000 simulations were performed with *O_tot_* randomly distributed among *L_tot_* sites. The significance of the sum of *G* scores (Fig. S1) was evaluated by the corresponding *z* score = (the observed sum − mean of simulated sums)/(standard deviation of simulated sums). To define the set of genes that overrepresented the candidate nonsynonymous mutations, for each gene *i*, we calculated the *P* value based on the proportion of simulated *G_i_* larger or equal to the observed *G_i_*. Multiple test correction then was performed by multiplying each gene’s *P* value by the number of genes with at least one candidate nonsynonymous mutation (Bonferroni correction). Only the genes with Bonferroni corrected *P* value of  <0.05 are called significant. We performed enrichment tests of gene ontology (GO) terms and KEGG pathways for this set of significant genes using the R package DOSE (80) with the functions “enrichGO” and “enrichKEGG,” a *q* value cutoff of 0.05, and the organismal database org.EcK12.eg.db.

10.1128/mbio.03467-21.3 FIG S3 Dependence of sum of G scores on the number of observed mutations. The null expectation of mutational parallelism (measured by the sum of G scores across genes) varies with the total number of mutations considered. The dashed line and dotted lines summarize the mean and 95% range across 1,000 simulations of hypothetical number of fixed nonsynonymous mutations: 1, 50, 100, 500, 1,000, 1,500, 2,000, 2,500, 3,000, 4,000, 5,000, 6,000, 7,000, 8,000, 9,000, and 10,000. Download FIG S3, PDF file, 0.06 MB. Copyright © 2022 Behringer et al. 2022 Behringer et al. https://creativecommons.org/licenses/by/4.0/ This content is distributed under the terms of the Creative Commons Attribution 4.0 International license.

For each resource-replenishment cycle/genetic background combination, we also tested whether a gene overrepresents the candidate structural mutations (indels and IS element insertions). The genic *G* score was similarly defined as the one for nonsynonymous mutations, while *O_i_* is the observed number of populations with any structural mutations in gene *i* and *L_i_* is the gene length for gene *i*. We also finished 20,000 simulations and calculated the Bonferroni corrected *P* value for each gene. As a result, we found all the genes with *O_i_* of ≥2 show Bonferroni corrected *P* value of <0.05.

### Calculation of mean Bray-Curtis similarity.

For each resource-replenishment cycle/genetic background combination, we also calculated the mean Bray-Curtis similarity across all pairs of experimental populations to quantify the parallelism among the candidate nonsynonymous mutations (44). Specifically, for a pair of populations, *j* and *k*, the Bray-Curtis similarity is calculated by

$$1-\frac{\Sigma_{i}\left|o_{\mathrm{ij}}\;-\;o_{\mathrm{ik}}\right|}{\Sigma_{i}\left(o_{\mathrm{ij}}\;+\;o_{\mathrm{ik}}\right)}$$

where *o_ij_* and *o_ik_* are the observed numbers of candidate nonsynonymous mutations in gene *i* for populations *j* and *k*, respectively. For each resource-replenishment cycle/genetic background combination, we performed 1,000 simulations to acquire the null distribution of mean Bray-Curtis similarity. In each simulation, we randomly distributed the number of candidate nonsynonymous mutations among *L_tot_* sites (double hits are not allowed) for each population. After acquiring the null distribution, we evaluated the significance of the mean Bray-Curtis similarity (Fig. S1) by corresponding *z* score = (the observed value − mean of simulated values)/(standard deviation from simulated values).

### Mutation rate estimation.

We performed fluctuation tests as described previously (40) to measure the mutation rates of our evolved populations (900-day samples) and their ancestors. Specifically, the resistance to the antimicrobial rifampin (0.01%, wt/vol) that is conferred by mutations to *rpoB* was analyzed. For each resource-replenishment cycle/genetic background combination, we studied four *ara* mutant populations with four independent clones for the first two populations and two independent clones for the last two populations. Two independent clones were used for the WT or MMR^−^ ancestor as well. For each clone, we performed 40 replicate experiments. We then determined the CFU counts/mL for all replicates, calculated the mean mutation rate, and estimated the corresponding 95% confidence interval using the R package rSalvador (81) with the function “newton.LD.”

### Determination of selection rate under 10-day feast/famine conditions.

Relative selection rate of evolved clones and populations was measured using a competitive coculture framework. Because our cultures were observed to occupy spatially distinct locations within the culture tubes, competitions to be assessed at day 1 (*T*_1_), day 4 (*T*_4_), and day 10 (*T*_10_) occurred in independent tubes for not disturbing any ecologically relevant structures. Evolved and ancestor clones were revived from −80°C storage by streaking for isolation on LB agar plates and incubating overnight at 37°C. Before competition, clones, ancestors, and populations were preconditioned to the competition environments either by inoculating a single isolate colony into 16- by 100-mm glass culture tubes containing 10 mL of LB broth for clones and ancestors or by inoculating a scrape of preserved frozen culture into a 16- by 100-mm glass culture tube for population samples. Preconditioning occurred over 24 h, with shaking upright on a rotator table at 175 rpm at 37°C. After 24 h of preconditioning, cell density of fully vortexed cultures was normalized to the ancestor based on absorbance at 600 nm. Once normalized, competitions were started by inoculating 16- by 100-mm glass culture tubes containing 10 mL of LB broth with 50 μL of ancestor culture and either 50 μL of evolved clone/population culture for single clone/population competition or 25 μL of each clone for double clone competition. Once competition cultures were mixed, culture tubes were fully vortexed, and 100 μL of *T*_0_ coculture was removed for CFU counting before incubating the competition tube with shaking upright on a rotator table at 175 rpm at 37°C. Coculture competition then occurred during incubation for either 1, 4, or 10 days before fully vortexing the culture tubes and removing 100 μL of coculture for CFU counting. Culture for CFU counting was prepared by serial dilution in 1× PBS before plating 100 μL of serially diluted culture on tetrazolium (TA) plus arabinose agar and incubated overnight at 37°C. Plating of culture on TA plus arabinose agar turns WT colonies pink and AraBAD^−^ colonies red for easy enumeration of genotypes. Selection rate (*r*) was determined as the difference of the natural logs of the ratio of each competitor CFU counts at the final and initial time points: *r* = ln(Evolved_TF_/Evolved_T0_) − ln(Ancestor_TF_/Ancestor_T0_) (49).

To test for negative frequency-dependent selection between the two ecotypes, we inserted the *ΔsrlD*780::*kan* marker from the Keio Collection line JW2674 into ecotype B via P1 transduction. Ecotype A and ecotype B were then competed against each other as described above, except that they were pooled to create different initial frequencies (ranging from ~90% ecotype A/10% ecotype B to ~90% ecotype B/10% ecotype A). Cultures were plated on TA plus sorbitol agar, which turns WT colonies pink/dusty rose and SrlD^−^ colonies red for easy enumeration of genotypes.

### RNA extraction, sequencing, and analysis.

RNA was isolated from two replicates of the WT ancestor and a representative clone from ecotype A and ecotype B. Growth from all samples was quenched at the relative mid-log-phase and at 36 h from LB broth cultures using the E.Z.N.A. bacterial RNA kit (Omega Bio-Tek). Mid-log phase was determined as the time point associated with maximum growth rate in LB as assessed from a 96-well microplate reader growth assay (Fig. S2A). We selected 36 h for the late-stationary-phase time point, as >90% of WT ancestor cells are still present in a live state with noncompromised membranes after quantification on an LSRII benchtop flow cytometer (BD) of cells stained with a LIVE/DEAD BacLight bacterial viability kit (L7012; Invitrogen) (Fig. S2B). RNA was submitted to the Genomics Core at the Biodesign Institute–Indiana University for rRNA depletion, library preparation, and sequencing on an Illumina NextSeq500 75-cycle, high-output run. RNA sequencing reads were preprocessed using Cutadapt v.1.9.1 (77) to remove residual adapters and trim low-quality sequences. Processed reads were then mapped to the Escherichia coli K-12 MG1655 reference genome (NCBI accession no. NC_000913.3) with HISAT2 v.2.1.0 with the –dta and –no-spliced-alignment options (82). Resulting SAM files were converted to bam format with SAMtools v.1.5 before annotating and quantifying transcripts with StringTie v.1.3.3b (83). To perform differential expression analysis, transcript abundances from StringTie were imported into R using tximport (countsFromAbundance = “lengthScaledTPM”) and analyzed with DESeq2, which uses a Wald’s test to determine significance of differential expression (84). To identify DEGs due to 10-day feast/famine cycles, transcript abundances from each ecotype were treated as biological replicates and compared to the WT ancestor. DEGs with an adjusted *P* value of <0.05 were considered significant and used for downstream GO analysis with DAVID v. 6.8 using the GOTERM_BP_DIRECT output. To identify DEGs between ecotypes, we calculated the ratio of normalized transcript counts for ecotype A versus ecotype B at mid-log phase and 36 h. Genes with a mean normalized transcript count of <25 across both ecotypes within a time point were removed from further analysis. Ratios of normalized transcript counts of  >1.5 were considered to have higher expression in ecotype A, while ratios of <−1.5 were considered to have higher expression in ecotype B. GO analysis was conducted with DAVID v. 6.8 using the GOTERM_BP_DIRECT output, and essential genes were identified using the list of genes in which deletion results in no growth in LB broth according to the EcoCyc database.

### Data availability.

All code necessary to repeat differential expression analysis can be found at (https://github.com/LynchLab/ECEE_Starvation), and all RNA sequencing reads can be downloaded from SRA at NCBI (BioProject number PRJNA532905).
