# Supplementary figures and images for: Complex Ecotype Dynamics Evolve in Response to Fluctuating Resources
Source: mBio. 2022 May 16;13(3):e03467-21. doi: 10.1128/mbio.03467-21 (PMC9239185; doi:10.1128/mbio.03467-21)

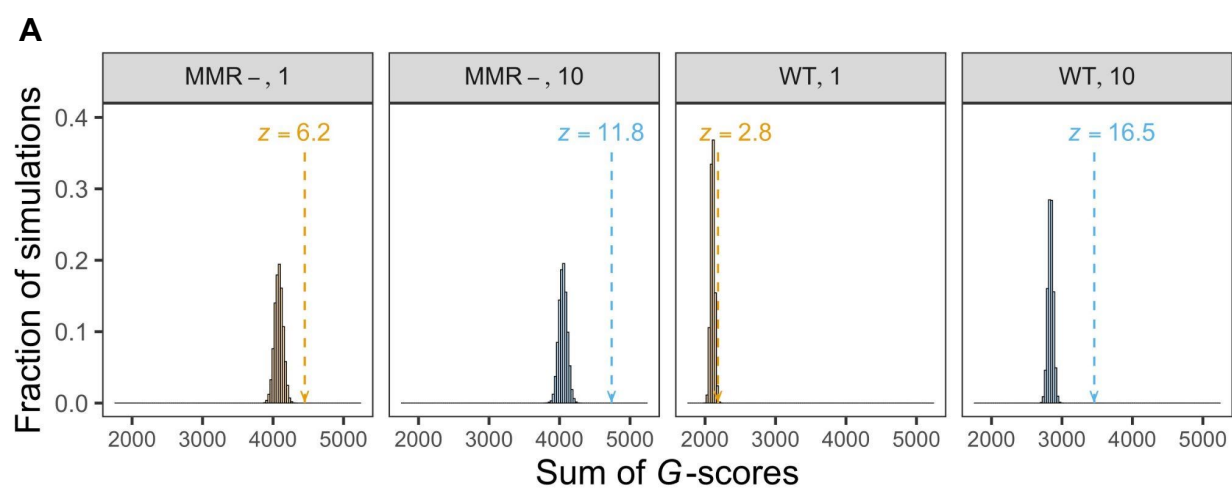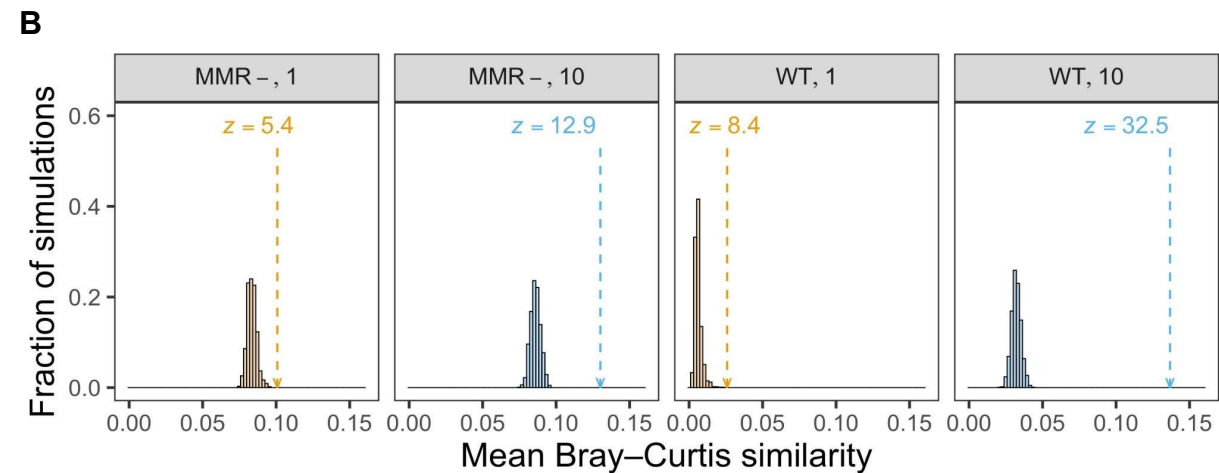

Supplement: FIG S1 [file mbio.03467-21-s0001.pdf]

**A**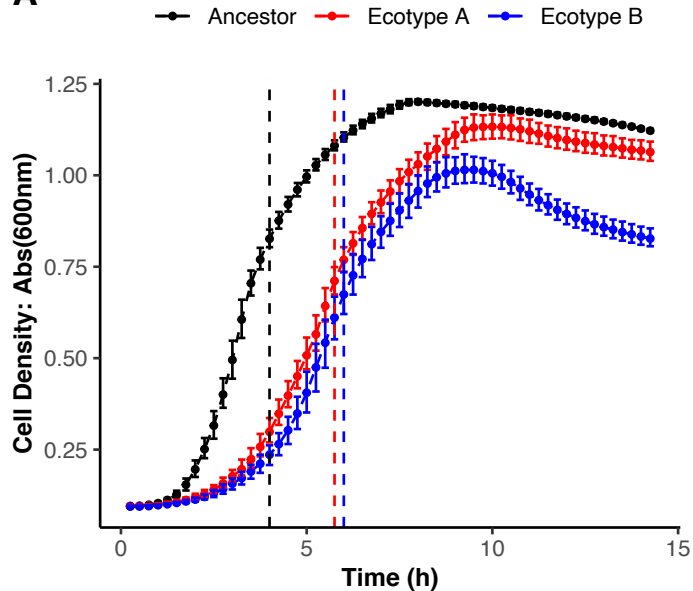**B**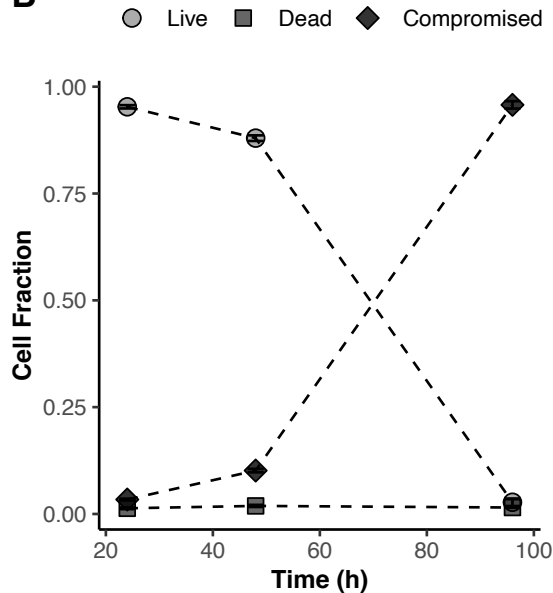

Supplement: FIG S2 [file mbio.03467-21-s0002.pdf]

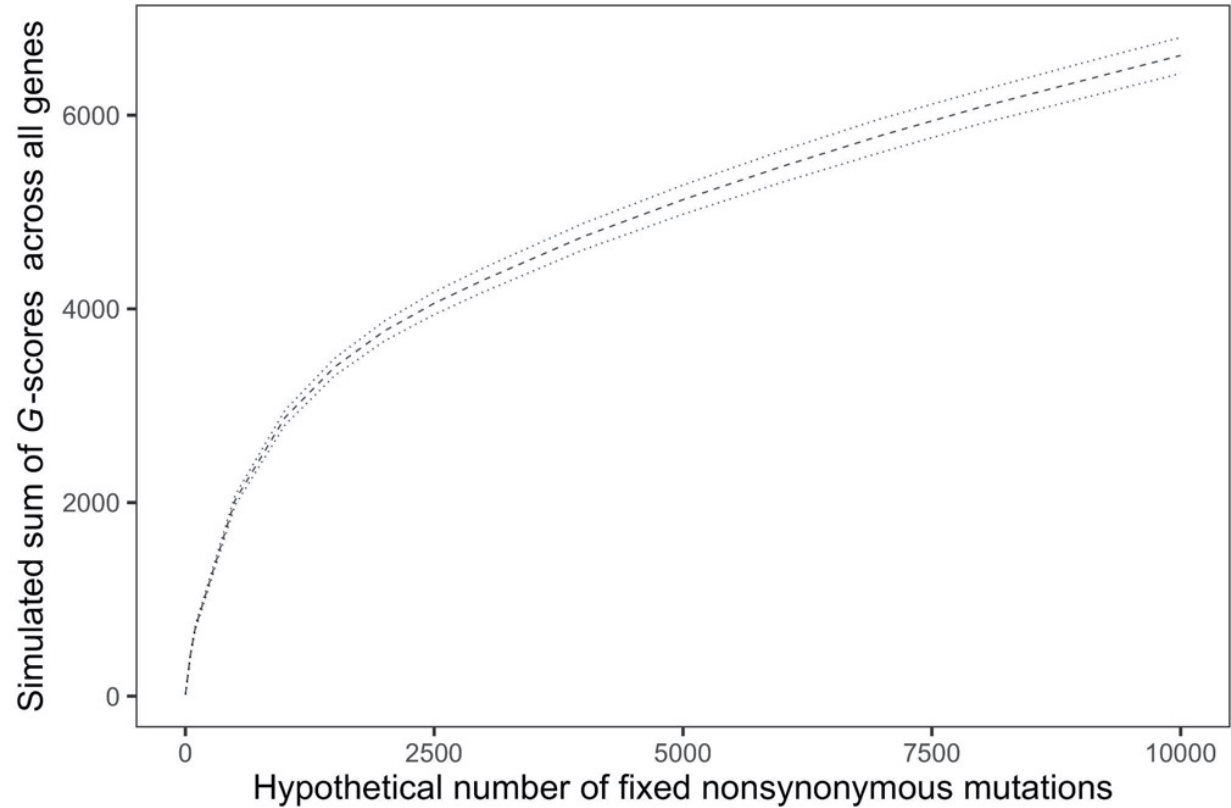

Supplement: FIG S3 [file mbio.03467-21-s0003.pdf]
